# Phylogeographic evidence for the inter- and intracontinental dissemination of avian influenza viruses via migration flyways

**DOI:** 10.1371/journal.pone.0218506

**Published:** 2019-06-26

**Authors:** Junki Mine, Yuko Uchida, Kirill Sharshov, Ivan Sobolev, Alexander Shestopalov, Takehiko Saito

**Affiliations:** 1 Division of Transboundary Animal Disease, National Institute of Animal Health, National Agriculture and Food Research Organization, Tsukuba, Ibaraki, Japan; 2 Thailand–Japan Zoonotic Diseases Collaboration Center, Kasetklang, Chatuchak, Bangkok, Thailand; 3 Federal Research Center of Fundamental and Translational Medicine, Novosibirsk, Russia; 4 United Graduate School of Veterinary Sciences, Gifu University, Gifu, Japan; St. Jude Children’s Research Hospital, UNITED STATES

## Abstract

Genetically related highly pathogenic avian influenza viruses (HPAIVs) of H5N6 subtype caused outbreaks simultaneously in East Asia and Europe—geographically distinct regions—during winter 2017–2018. This situation prompted us to consider whether the application of phylogeographic analysis to a particular gene segment of AIVs could provide clues for understanding how AIV had been disseminated across the continent. Here, the N6 NA genes of influenza viruses isolated across the world were subjected to phylogeographic analysis to illustrate the inter- and intracontinental dissemination of AIVs. Those isolated in East Asia during winter and in Mongolia/Siberia during summer were comingled within particular clades of the phylogeographic tree. For AIVs in one clade, their dissemination in eastern Eurasia extended from Yakutia, Russia, in the north to East Asia in the south. AIVs in western Asia, Europe, and Mongolia were also comingled within other clades, indicating that Mongolia/Siberia plays an important role in the dissemination of AIVs across the Eurasian continent. Mongolia/Siberia may therefore have played a role in the simultaneous outbreaks of H5N6 HPAIVs in Europe and East Asia during the winter of 2017–2018. In addition to the long-distance intracontinental disseminations described above, intercontinental disseminations of AIVs between Eurasia and Africa and between Eurasia and North America were also observed. Integrating these results and known migration flyways suggested that the migration of wild birds and the overlap of flyways, such as that observed in Mongolia/Siberia and along the Alaskan Peninsula, contributed to the long-distance intra- and intercontinental dissemination of AIVs. These findings highlight the importance of understanding the movement of migratory birds and the dynamics of AIVs in breeding areas—especially where several migration flyways overlap—in forecasting outbreaks caused by HPAIVs.

## Introduction

Influenza A viruses have been isolated from birds and from various mammals, including pigs and humans [1]. Wild waterbirds (e.g., Anseriformes and Charadriiformes) form the natural reservoir of avian influenza viruses (AIVs), which retain H1–H16 hemagglutinin and N1–N9 neuraminidase [2–6]. AIVs often spread to geographically distinct regions or across major water bodies, or both, as wild birds migrate, because AIV infection usually does not cause clinical signs in these birds or disturb their long-distance migrations [7, 8]. In fact, phylogenetic analyses of whole genomes revealed that AIVs carrying genes of the North American lineage were isolated in Europe, and AIVs carrying genes of the Eurasian lineage were isolated in the United States [9–14].

The isolation of highly pathogenic avian influenza viruses (HPAIVs) from wild birds was rare [15, 16] until 1996, when the situation changed with the emergence of H5N1 HPAIVs that caused outbreaks among domestic geese in China [17]. The first outbreak caused among wild birds by H5N1 HPAIVs was recognized in multiple bird species in Hong Kong in 2002 [18]; this was followed by a die-off of Anseriformes and Charadriiformes in Qinghai Lake in April 2005 [19] and isolation of the viruses—so-called Qinghai strains—from wild birds in western Siberia in July 2005 [20]. The strains spread to Europe and West Africa between October 2005 and February 2006 [21, 22]. The movement of migratory birds, as well as the poultry trade, played an important role in the spread of these viruses [21, 23, 24]. During the winter of 2014–2015, H5 HPAIVs related to the viruses circulating in Asia spread to North America [25, 26]. Some studies suggested that the movement of migratory birds from Asian wintering sites to breeding sites in Far East Siberia and the Alaskan Peninsula, and the subsequent southward movement along the Pacific coast of North America, could be related to this spread of HPAIVs [27, 28]. In addition, a previous report demonstrated that H5 HPAIVs, which showed high pathogenicity in poultry under experimental infection, caused mild or no clinical signs in wild birds [29]. These events highlight the involvement of wild birds in the spread of AIVs, including HPAIVs [30].

During the winter of 2017–2018, both Asia and Europe experienced outbreaks of disease caused by H5N6 HPAIVs [31]. Several reports demonstrated that H5N6 HPAIVs isolated at a duck farm in the Netherlands in December 2017 [32] were genetically related to H5N6 HPAIVs isolated from poultry in North Jeolla Province, Korea, in November 2017 [33]. They were considered to have emerged through several re-assortments between H5N8 HPAIVs causing outbreaks in 2016–2017 and HxN6 AIVs [33–35].

In this study, we performed a phylogeographic analysis of N6 NA genes deposited in public databases and our institutional repositories, including the H5N6 HPAIVs isolated during the winter of 2017–2018, to illustrate intra- and intercontinental dissemination of AIVs and to assess the relationship between virus dissemination and migration flyways of wild birds. Our findings should contribute to the strategic monitoring of AIVs in wild birds as part of efforts to clarify the ecology of AIVs and forecast HPAI outbreaks.

## Materials and methods

### Ethics statement

The activities on sampling from wild birds in Russia were approved by the Committee on Biomedical Ethics of the Federal Research Center of Fundamental and Translational Medicine (No. 2017–16). No specific permission was required for sample collection from wild birds killed by local hunters in compliance with the Russian Federation hunting laws. Also, no permit was needed for sampling from wild birds captured alive during ringing activities in Biostations with collaboration with other research institute as part of the Russian national avian influenza surveillance. Sampling in Japan for viruses in this study required no permission as they are feces collected under the avian influenza surveillance of the Ministry of the Environment or samples applied for the diagnosis of avian influenza in National Institute of Animal Health in Japan. Samples from Vietnam and Cambodia are also not applicable as they originated to poultry and were collected for diagnosis.

### Virus isolation and whole-genome sequencing

Nasal or cloacal swabs, or both, and feces were collected from poultry and wild birds in Japan, Vietnam, and Cambodia from 1977 to 2018 and preserved in media as described previously [36]. At the National Institute of Animal Health in Japan, samples were inoculated into the allantoic cavities of 10- to 11-day-old embryonated chicken eggs and incubated for 24–48 h at 37 °C for virus isolation. The hemagglutination (HA) activity of allantoic fluid was tested by using 0.55% chicken red blood cells. A/duck/Japan/AnimalQuarantine-HE72/2015 and A/chicken/Japan/AnimalQuarantine-HE144/2016 originating in poultry meats that were illegally transported into Japan [37] were isolated by the Japanese animal quarantine service.

The whole genomes of the isolated viruses were obtained by using next-generation sequencing (Miseq, Illumina, San Diego, CA, USA). RNA was extracted from isolated viruses by using an RNeasy Mini kit (Qiagen, Hilden, Germany). cDNA libraries for next-generation sequencing were prepared by using an NEBNext Ultra RNA Library Prep Kit for Illumina (New England Biolabs, Ipswich, MA, USA). A total of 10 pM of synthesized cDNA libraries was mixed with 10 pM of PhiX control (Illumina) and sequenced by using a Miseq Reagent Kit (version 2, Illumina). The consensus sequences were generated by using Workbench software (version 9.5.3, Qiagen) or FluGAS software (version 1.0.0, World Fusion, Tokyo, Japan). The N6 NA sequences of the viruses isolated in this study have been deposited in the GISAID database (http://platform.gisaid.org); accession numbers of each virus are listed in S1 Table.

### Phylogenetic and phylogeographic analysis

For phylogenetic analysis, we downloaded sequences of the N6 NA genes from the GISAID database in January 2019. Sequences of the AIVs that the National Institute of Animal Health possessed (S1 Table) and of those isolated in Russia that the Federal Research Center of Fundamental and Translational Medicine possessed were aligned with the sequences downloaded from GISAID by using BioEdit [38] and MAFFT [39]. After the alignment, a total of 3720 sequences were used in the phylogenetic analysis performed by using MEGA-CC with 1000 bootstrap replicates according to the maximum likelihood method in a general time-reversible model [40].

The location-annotated maximum clade credibility (MCC) trees for selected clusters of the maximum likelihood tree (see Results) were constructed according to Bayesian stochastic search variable selection by using the Bayesian Evolutionary Analysis by Sampling Tree package version 1.8.2 [41], as described previously [42]. Asymmetric substitution model with Bayesian stochastic search variable selection and a strict clock model were applied for calculating Bayes factors in the present analysis. Then the output tree was visualized by spatial phylogenetic reconstruction of evolutionary dynamics using data-driven documents (SPreaD3) version 0.9.7 [43]. Lines with Bayes factors of 3.0 or more were indicated in each map.

### Correlation analysis between traits and phylogeny

Identical sequences of strains isolated from the same species, on the same date, and in the same place were removed from the N6 NA sequence alignment, and MCC trees without information about collection time were constructed. Calculations of constructed MCC trees were set as 1 × 10^7^ steps long to generate 10,000 trees, and the last 1000 trees were then used for Bayesian tip-association significance testing (BaTS) [44] to evaluate the correlation between traits such as host order and subtypes of isolated viruses and phylogeny. *P* values < 0.05 were used as evidence to support correlations between traits and tree topology.

## Results

### Clade definition of N6 NA genes

**Fig 1:**
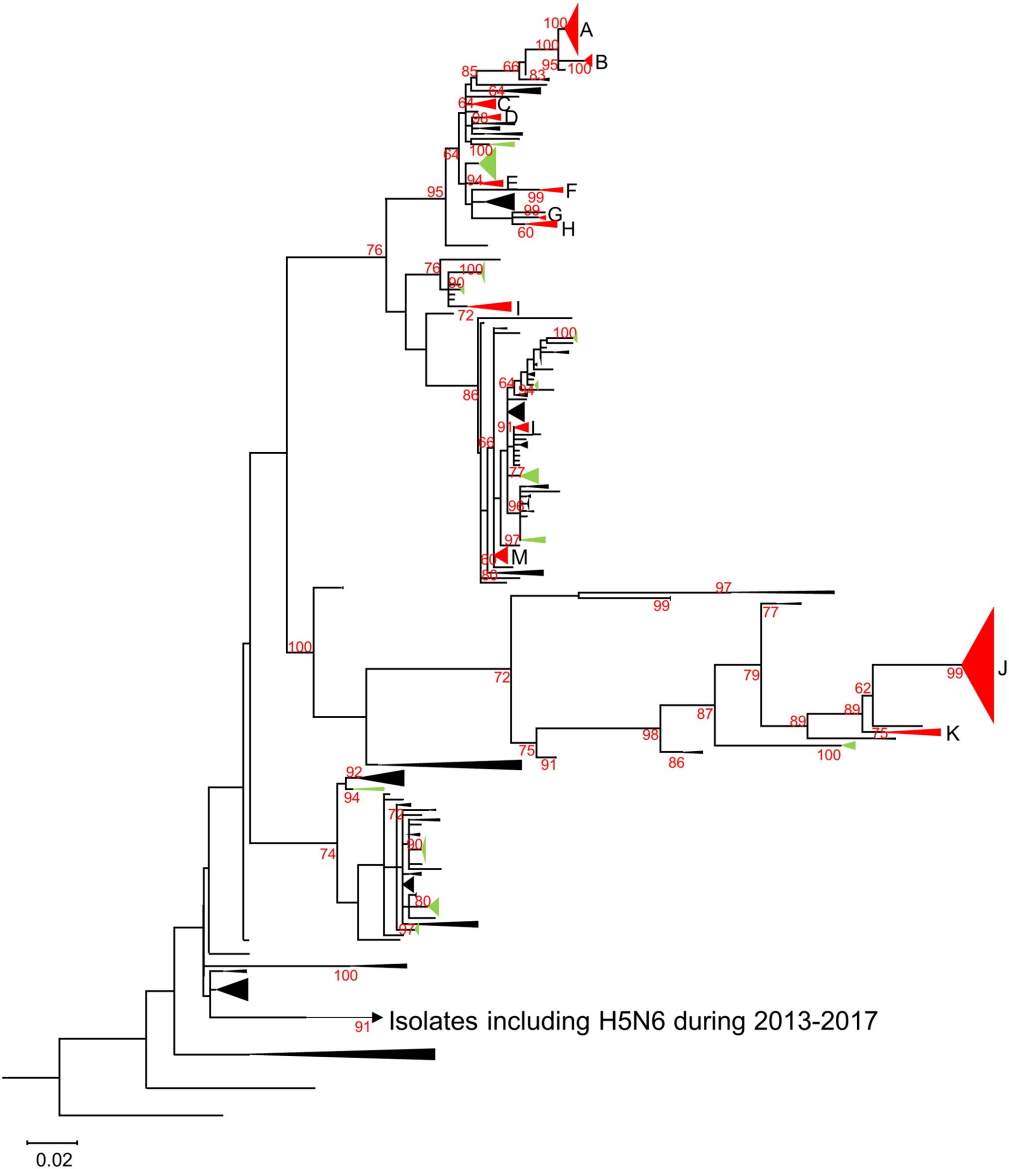
Entire N6 NA maximum likelihood phylogenetic tree composed of genes of viruses analyzed in this study and downloaded from the GISAID databases. AIVs classified as the Eurasian lineage are shown. Identified clades defined on the basis of the rule in this study are in green and red; red clades were selected for phylogeographic analysis and correlation analyses of bird host species and subtypes with phylogeny.

**Fig 2:**
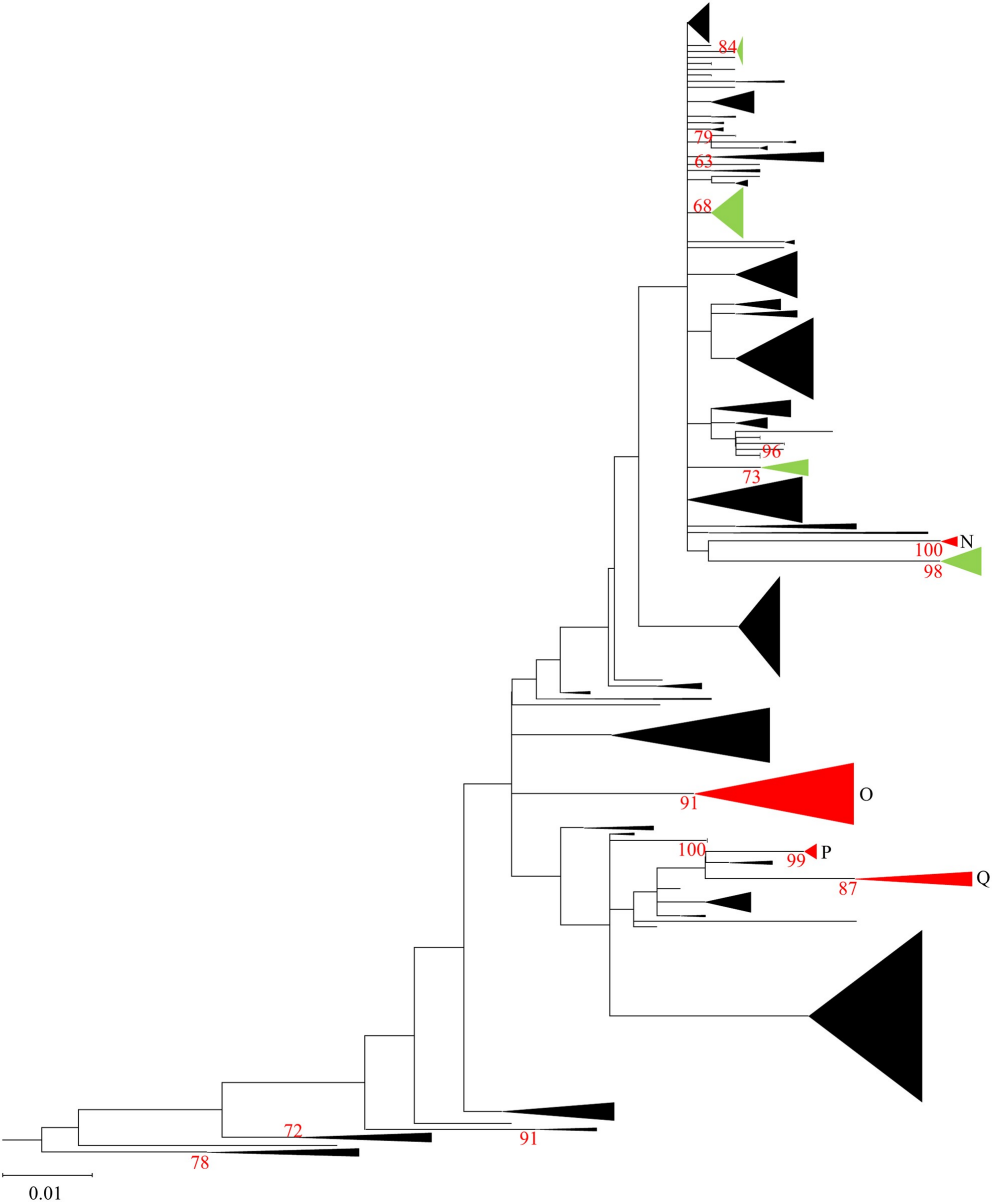
Entire N6 NA maximum likelihood phylogenetic tree composed of genes of viruses analyzed in this study and downloaded from the GISAID databases. AIVs classified as the North American lineage are shown. Identified clades defined on the basis of the rule in this study are in green and red; red clades were selected for phylogeographic analysis and correlation analyses of bird host species and subtypes with phylogeny.

To elucidate the intra- and intercontinental dissemination of genetically related AIVs by using phylogeographic analysis, a phylogenetic tree was constructed on the basis of the N6 NA genes of AIVs isolated worldwide. Phylogenetic analysis revealed that the N6 NA genes could be categorized into an Eurasian (Fig 1) and North American (Fig 2) lineage lineage, with a bootstrap value of 60 or more. By applying the criteria of the clade definition of H5 HA genes of Eurasian HPAIV [45], eight and 25 clades that consisted of at least five strains were recognized in the North American and Eurasian lineages, respectively (Figs 1 and 2, S1–S7 Figs). Among these, 17 clades (clades A to Q) were subjected to phylogeographic analysis because they consisted of strains originating from two or more countries (or states and provinces in the North American lineage) and were isolated in several years.

### Dissemination of N6 NA genes across the Eurasian continent

**Fig 3:**
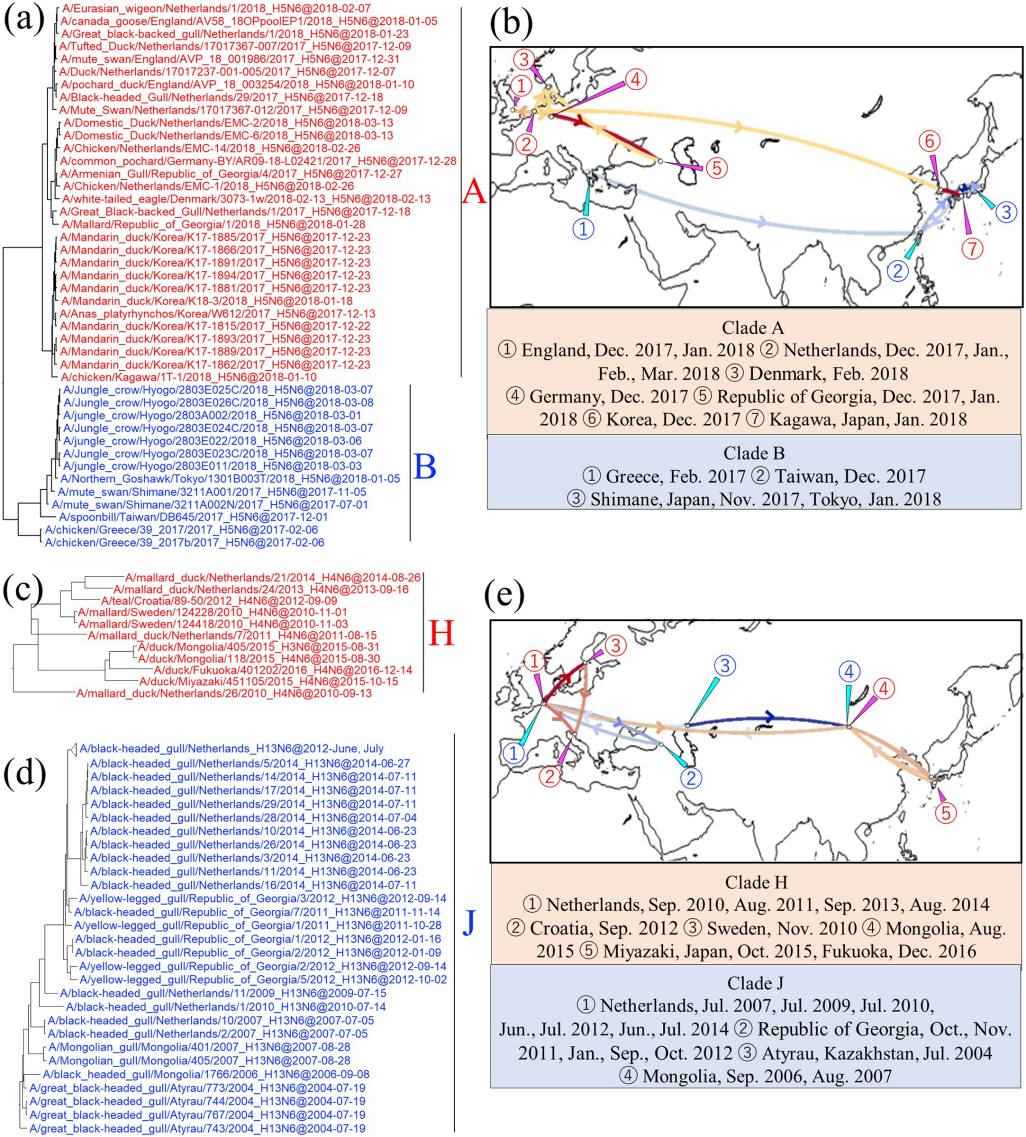
Detail of maximum clade credibility (MCC) phylogenetic trees of N6 NA genes in clades A, B, H, and J and visualized location-annotated MCC trees for selected clades on the world map. Clades A and B are red and blue, respectively, in tree (a), and colored type lines in (b) correspond to the clade colors. Clades H and J are red type and blue type, respectively, in each tree (c and d), and colored type lines in (e) correspond to those of clades. Lines with Bayes factors of 3.0 or more are shown in the map, and a deeper color means a higher Bayes factor.

N6 NA genes of the H5N6 HPAIVs isolated in Asia and Europe during the winter of 2017–2018 were categorized into two clades, A and B (Fig 3a, S1 Fig), in the Eurasian lineage. The genes were significantly distinct from those of H5N6 HPAIVs that were enzootic in Asia before the 2017–2018 season (Fig 1), showing nucleotide identities with them of 83.8% and 84.1%, respectively. Clade A was composed of H5N6 HPAIVs isolated in East Asia, western Asia, and Europe; those isolated in East Asia formed a subcluster and those in western Asia and Europe formed another in the clade (Fig 3a). Clade B was composed of those isolated in East Asia forming cluster B, along with isolates from poultry in Greece collected in February 2017 (Fig 3a). The identity between clades A and B was 97.3%. H3N6 AIVs isolated in the Netherlands in December 2014 and in Novosibirsk, Russia, in September 2017 were classified as an outgroup to clades A and B (S1 Fig).

The spatial and temporal relationships of clades A and B were phylogeographically analyzed by calculating the Bayes factors between the locations where AIVs were isolated (S2 Table). The Bayes factor from the Netherlands to Korea was 4.77, and that from the Netherlands to the Republic of Georgia was 146.42, indicating the strong relationships among H5N6 HPAIVs in western and eastern Eurasia (Fig 3b red type lines). Considering the simultaneous dissemination to Europe and Asia in the same season, it is reasonable to assume that H5N6 HPAIVs had emerged in neither Europe nor Asia before they reached those areas. On the other hand, in clade B, descendant viruses that shared a common ancestor with Greek isolates were disseminated eastward and were detected in Japan and Taiwan in the winter of 2017–2018 (Fig 3b, blue type lines), although it is not plausible that Greek isolates were disseminated directly to Asia without any relay point after a year of absence.

Like clades A and B, clades H and J were composed of AIVs isolated in both Asia and Europe. In clade H, isolates collected in Sweden, the Netherlands, and Croatia from 2010 to 2014 were related to Mongolian isolates collected in August 2015 and to Japanese isolates in October 2015 and December 2016, with identity of 98.6% (Fig 3c). Phylogeographic analysis suggested that H4N6 AIVs that had been isolated in Europe crossed the Eurasian continent and reached Japan (Fig 3e, red type lines). This movement is similar to that observed in clade B, where H5N6 HPAIVs phylogenetically related to the isolates collected in Greece in February 2017 were isolated in Japan and Taiwan after the latter half of 2017, although the collection times of the Japanese and Dutch isolates in clade H differed by more than a year (Fig 3b, blue type lines). Clade J was also composed of AIVs isolated in East Asia, central Asia, western Asia, and Europe, with an identity of 98.9% within the clade (Fig 3d). In this clade, AIVs were disseminated between East Asia and Europe, as an isolate collected in Atyrau, Kazakhstan, in July 2004 was related to AIVs isolated in Mongolia in September 2006 and September 2007 and in the Netherlands from July 2007 onward (Fig 3e, blue type lines).

### Intracontinental dissemination of N6 NA genes within Asia, Europe, or North America

**Fig 4:**
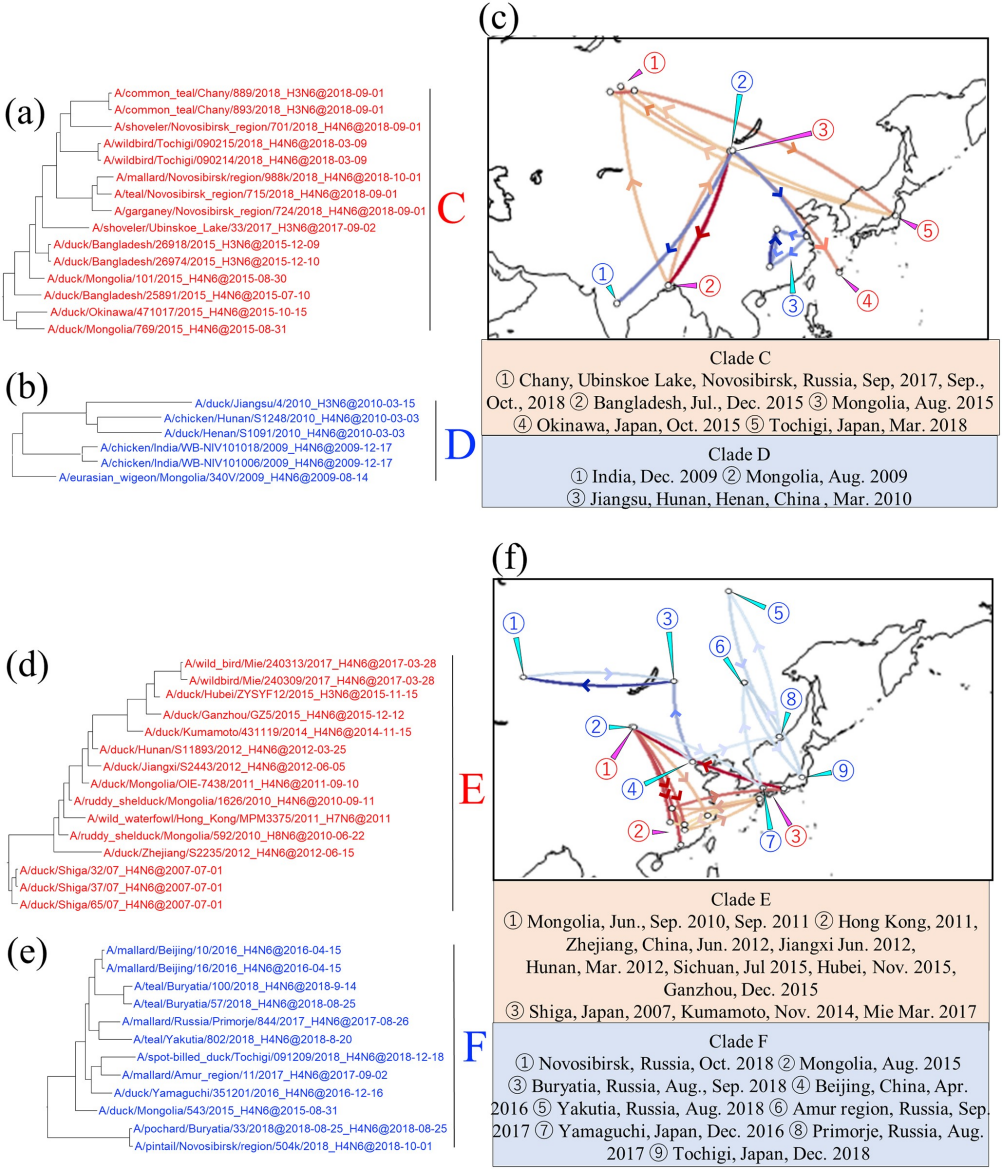
Detail of maximum clade credibility (MCC) phylogenetic trees of N6 NA genes in clades C, D, E, and F and visualized location-annotated MCC trees for selected clades on the world map. Clades C and D are red type and blue type, respectively in each tree (a and b), and colored type lines in (c) correspond to those of the clades. Clades E and F are red type and blue type, respectively, in each tree (d and e), and colored type lines in (f) correspond to those of the clades. Lines with Bayes factors of 3.0 or more are shown in the map, and a deeper color means a higher Bayes factor.

Clades C, D, E, and F were composed exclusively of AIVs isolated in Asia. AIVs isolated in Mongolia, the Asian part of Russia, Bangladesh, and Japan after 2011 formed clade C, showing identity of 98.5% within the clade (Fig 4a). Isolates collected in Novosibirsk during summer in 2017 and 2018 were significantly related to the isolates collected in Mongolia in 2015, Bangladesh in 2015, and Tochigi Prefecture, Japan, in 2018, and the Mongolian isolates were significantly related to the isolates in Bangladesh and Okinawa Prefecture, Japan, in 2015 (Fig 4c, red type lines). Clade D was composed of AIVs isolated in China, Mongolia, and India from August 2009 to March 2010, showing identity of 98.9% within the clade (Fig 4b and 4c, blue type lines). It might be the case that a descendant of A/eurasian wigeon/Mongolia/340V/2009_H4N6 was disseminated to India 4 months later. Clade E was composed of isolates gathered in Japan, China, and Mongolia from 2007 onward, with 98.6% identity within the clade (Fig 4d). In this clade, descendants of the isolates collected in Shiga Prefecture, Japan, in 2007 were found in China and Mongolia after 2010 and in Mie Prefecture, Japan, in March 2017 (Fig 4f, red type lines). Compared with the Bayes factors between Japan and China (average: 5.76), those between Japan and Mongolia and between Mongolia and China (average: 13.16) were significantly higher. AIVs isolated from East Asia from August 2015 to 2017 formed a unique clade, designated as clade F (Fig 4e, S2 Fig). The identity of the clade was as high as 98.7%, and the gene sequences differed by 3% or more from those of the other clades. The viruses in clade F were from Far East Russia, Yamaguchi and Tochigi prefectures, Japan, Beijing, China, and Mongolia. On the basis of the phylogeographic analysis, we postulate that descendants of the Japanese isolate collected in December 2016 appeared in Far East Russia (Amur region) in September 2017 and reappeared in Japan in December 2018 (Fig 4f, blue type lines). Along with this dissemination, a relationship between AIVs farther north in Primorje and Yakutia was also found.

**Fig 5:**
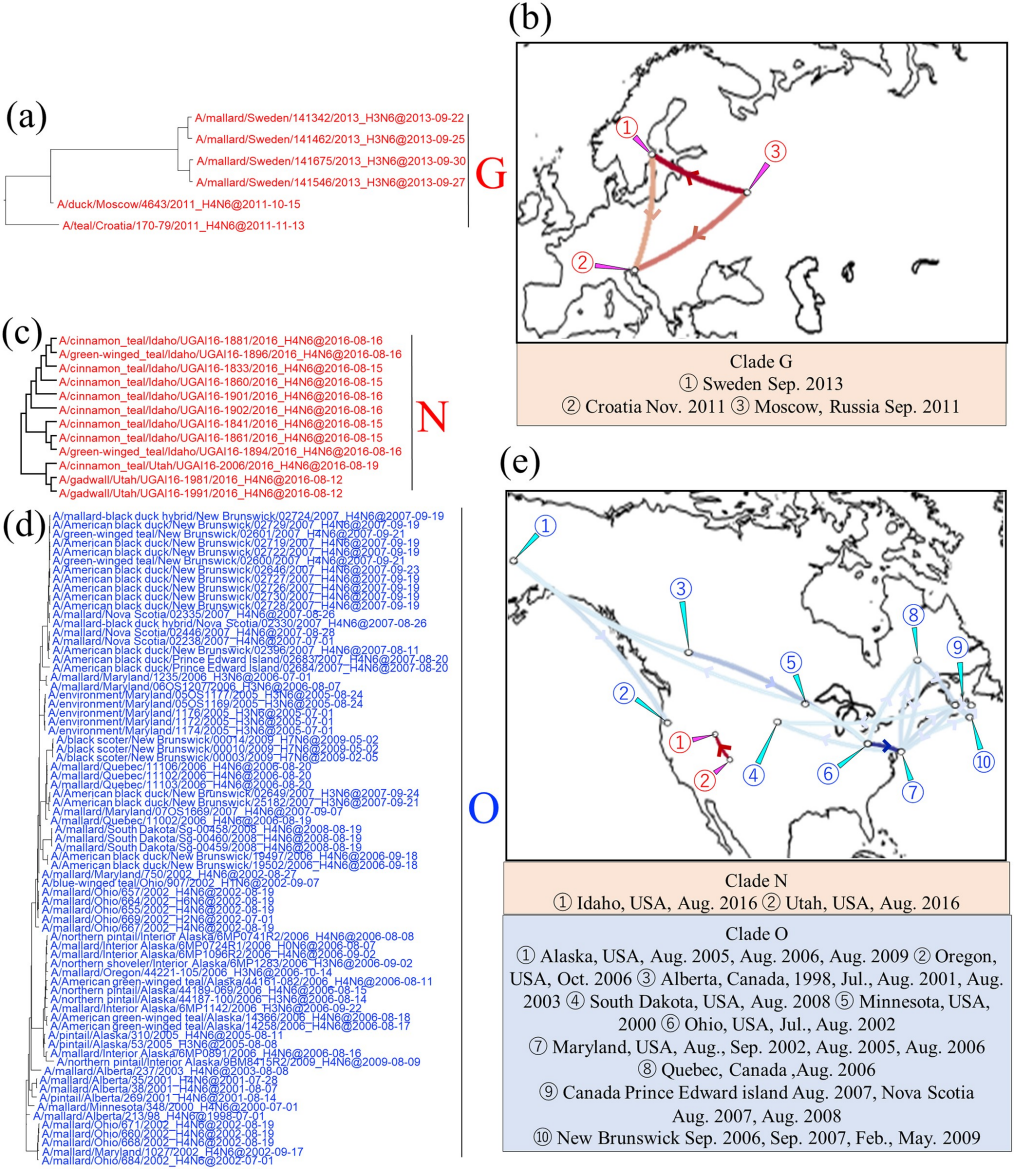
Detail of maximum clade credibility (MCC) phylogenetic trees of N6 NA genes in clades G, N, and O and visualized location-annotated MCC trees for selected clades on the world map. Clade G is red type in the tree (a), and colored type lines in (b) correspond to the clade. Clades N and O are red type and blue type, respectively, in each tree (c and d), and colored type lines in (e) correspond to those of the clade. Lines with Bayes factors of 3.0 or more are shown in the map, and a deeper color means a higher Bayes factor.

**Fig 6:**
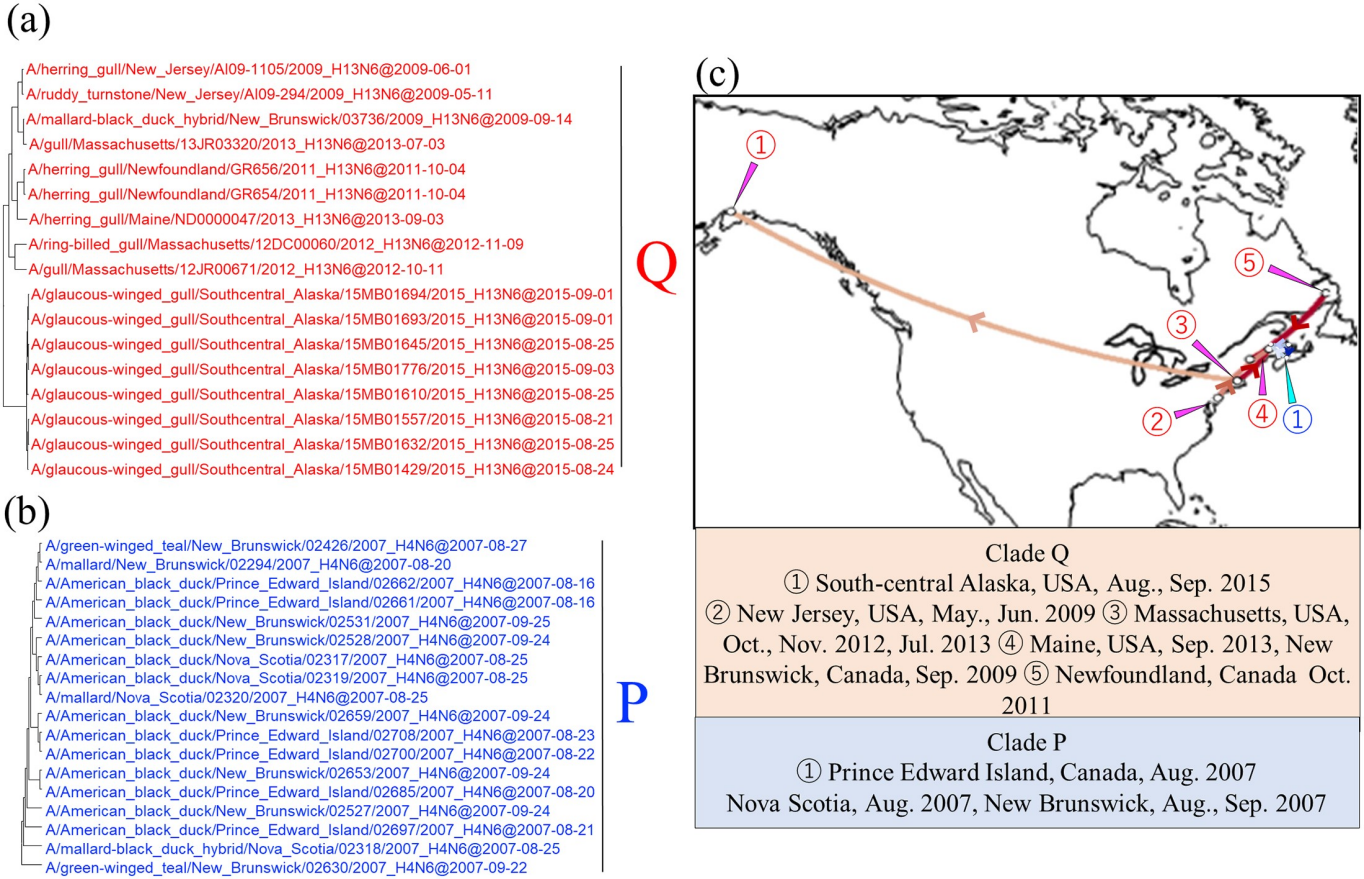
Detail of maximum clade credibility (MCC) phylogenetic trees of N6 NA genes in clades Q and P and visualized location-annotated MCC trees for selected clades on the world map (c). Clades Q and P are red type and blue type, respectively, in each tree (a and b), and colored type lines in (c) correspond to those of the clade. Lines with Bayes factors of 3.0 or more are shown in the map, and a deeper color means a higher Bayes factor.

Intracontinental movements of AIVs in Europe (clade G) or North America (clades N, O, P, and Q) were recognized. In clade G, AIVs isolated in Croatia in November 2011 and in Moscow, Russia, in October 2011, and those isolated in Sweden in September 2013, shared a common ancestor, with an identity of 99.3% (Fig 5a and 5b, red type lines). Clades N, O, P, and Q belonged to the North American lineage (Fig 2). More than 70 isolates collected from 1998 to 2009 across North America comprised Clade O, with an identity of 98.7% (Fig 5d and 5e, blue type lines). As with the case of clade O, clade Q was composed of AIVs isolated on the west and east coasts of the North American continent, with an identity of 98.6% (Fig 6a and 6c, red type lines). Clades N and P consisted of strains isolated from relatively small areas during brief periods. Isolates of clades N were collected from the neighboring states of Utah and Idaho in the United States in August 2016, with identity of 99.7% (Fig 5c and 5e, red type lines). Those of clade P were collected from Canadian provinces along the Atlantic Ocean (i.e., Prince Edward Island, Nova Scotia, and New Brunswick) in August and September 2007, with identity of 99.9% (Fig 6b and 6c, blue type lines).

### Intercontinental dissemination of N6 NA genes between Europe and Africa and between Eurasia and North America

**Fig 7:**
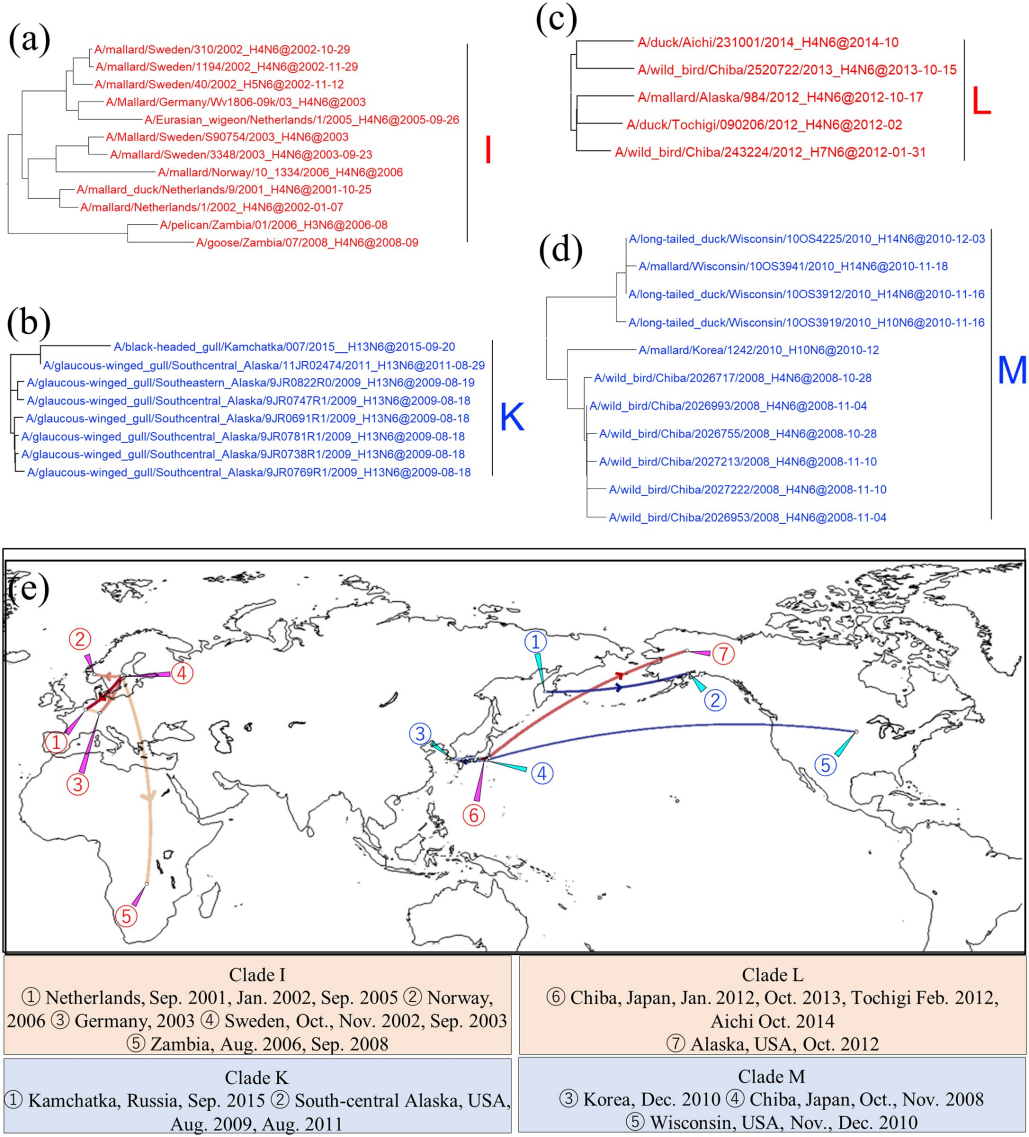
Detail of maximum clade credibility (MCC) phylogenetic trees of N6 NA genes in clades I, K, L, and M and visualized location-annotated MCC trees for selected clades on the world map (e). Clades I, K, L, and M are red type, blue type, red type, and blue type, respectively, in each tree (a, b, c, and d), and colored type lines in (e) correspond to those of the clades. Lines with Bayes factors of 3.0 or more are shown in the map, and a deeper color means a higher Bayes factor.

Intercontinental dissemination of N6 NA genes was observed in clades I, K, L, and M. Clade I consisted of AIVs isolated in Europe and Zambia, with identity of 98.7% within the clade (Fig 7a). AIVs isolated in Sweden, the Netherlands, Norway, and Germany from October 2001 to September 2005 shared a common ancestor with isolates collected in Zambia in 2006 and 2008 (Fig 7e, red type lines). One of the two Zambian strains was isolated from a pelican, a bird species that is rarely reported as carrying AIVs partially because pelicans have less become the target of surveillance. Clades K, L, and M consisted of isolates from Far East Asia and the United States. N6 NA genes of isolates collected from south-central Alaska in 2009 and 2011 were closely related to those collected from the Kamchatka Peninsula in 2015, with identity of 99.5% in clade K (Fig 7b and 7e, dark blue type line). Clade L was composed of Japanese and Alaskan strains (Fig 7c). Japanese isolates from 2012 to 2014 formed clade L, with 99.4% identity, along with an Alaskan isolate collected in 2012 (Fig 7e, red type lines). Isolates collected from Chiba Prefecture, Japan, in 2008 formed clade M with those sampled from Korea in December 2010 and from Wisconsin in November and December 2010, with an identity of 99.5% (Fig 7d and 7e, light blue type line).

### Relationship between host, HA subtype, and clades

Classification of clades by phylogenetic analyses revealed that some, such as clades J and K, were composed of specific subtypes or had specific host species. To investigate the statistically significant correlations between topology and subtype or host specificity, N6 NA sequence alignment was calculated for BaTS analysis (Table 1). There was host specificity in approximately half of all species in Anseriformes (33 of 62 species) and Charadriiformes (10 of 20 species). All the Charadriiformes harboring clade J (black-headed gull, yellow-legged gull, Mongolian gull, and great black-headed gull) and clade K (black-headed gull and glaucous-winged gull) were calculated to significantly cluster together in the phylogenetic tree. In the case of other orders, there was no significant host specificity in the Gruiformes, Pelecaniformes, and Galliformes (except chicken and quail), although there was in two species of Columbiformes (pigeon and turtledove).

**Table 1:** Correlation of bird species with phylogeny.

Order	Number of species	Number of P<0.05 species	P<0.05 species
Accipitriformes	4	0	
Anseriformes	62	33	American black duck
			Chilean teal
			American wigeon
			Bewicks swan
			duck
			domestic duck
			goose
			mallard
			muscovy
			mute swan
			northern pintail
			northern shoveler
			stellers eider
			black scoter
			black swan
			blue winged teal
			cinnamon teal
			common shelduck
			emperor goose
			gadwall
			gray teal
			green winged teal
			migratory duck
			migratory waterfowl
			mule duck
			pintail
			pochard
			redhead
			ruddy shelduck
			surf scoter
			tundra swan
			whooper swan
			yellow billed pintail
Artiodactyla	1	1	swine
Carnivora	3	2	Caspian seal
			feline
Charadriiformes	20	10	black headed gull
			great black headed gull
			Mongolian gull
			glaucous winged gull
			gull
			herring gull
			ruddy turnstone
			sanderling
			shorebird
			yellow legged gull
Columbiformes	2	2	pigeon
			turtledove
Falconiformes	1	1	peregrine falcon
Galliformes	7	2	chicken
			quail
Gruiformes	4	0	
Passeriformes	3	2	jungle crow
			oriental magpie robin
Pelecaniformes	3	0	
Podicipediformes	1	0	
Primate	1	0	
Psittaciformes	1	0	
Pterocidiformes	1	0	
Rodentia	1	1	muskrat
Strigiformes	3	0	

Of the 15 subtypes (H1–H14 and H16), 11 (H1N6, H3N6, H4N6, H5N6, H6N6, H7N6, H9N6, H10N6, H11N6, H13N6, and H14N6) were calculated to cluster together in a phylogenetic tree (data not shown). Such subtype specificity was depicted in H4N6 subtypes in clades F, N, and P and in H13N6 subtypes in clades K, J, and Q.

## Discussion

**Fig 8:**
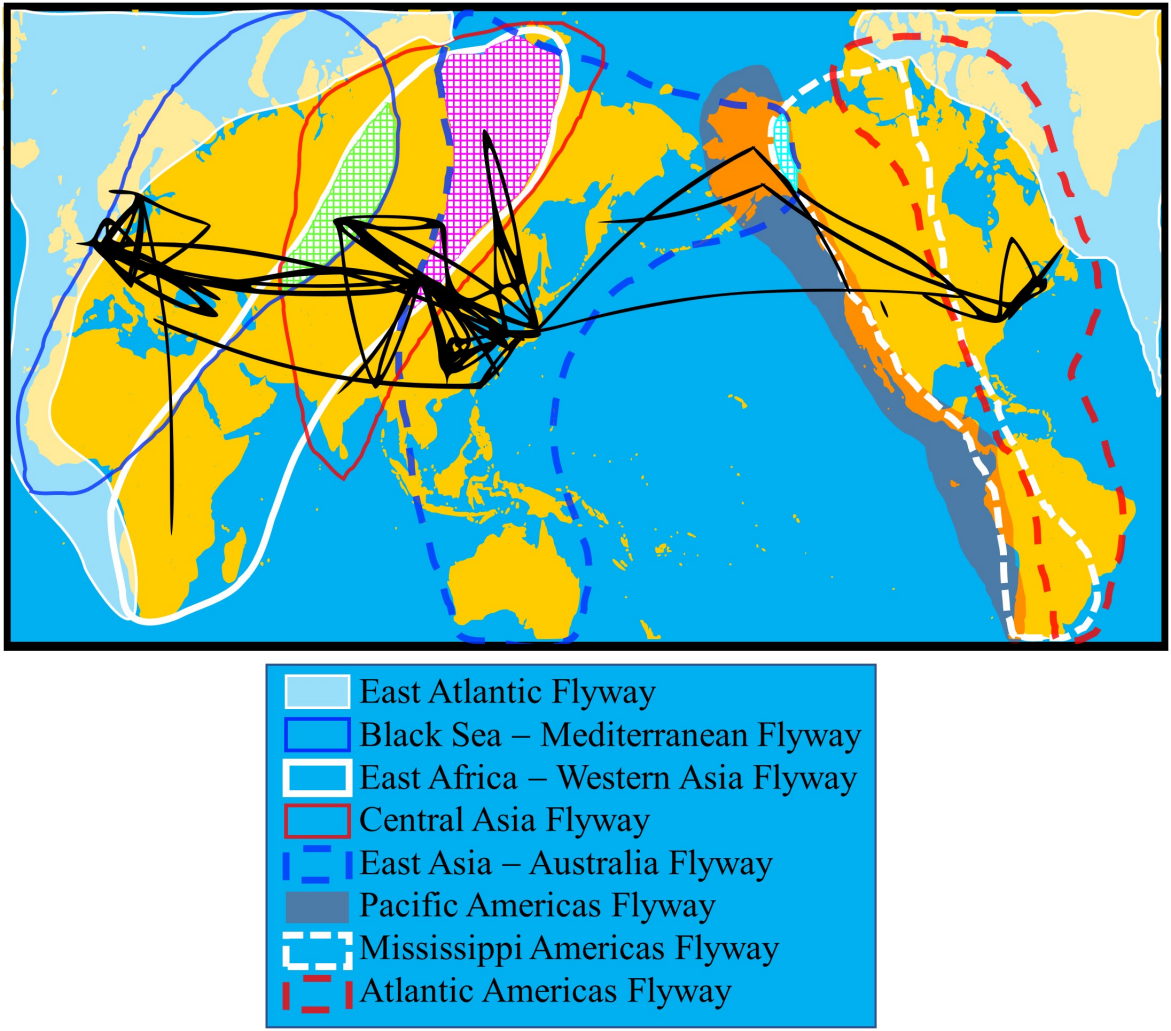
Visualization of relationship between movement of shorebirds and spread of avian influenza viruses. Location-annotated maximum clade credibility trees for all selected clades (A–Q) on a world map were merged with previously reported flyways of shorebirds [47]. Regions where 3 flyways overlap are shaded.

During the winter of 2017–2018, genetically closely related H5N6 HPAIVs caused disease outbreaks in Asia and Europe [31]. Our phylogeographic analysis of the N6 NA gene revealed that during that season the H5N6 HPAIVs in Asia and Europe diverged from a putative common ancestor and simultaneously reached both sides of the Eurasian continent. Considering that they were disseminated by wild birds, the H5N6 HPAIVs could have been disseminated from a breeding site, possibly in Siberia, where migratory birds following several flyways stretching across the Eurasian continent cohabitate during summer, although a direct ancestor could not be found (Fig 8) [46, 47]. Westward movement of HPAIVs from East Asia to Europe was recognized in the spread of the Qinghai strains from 2005 to 2006 [19–22], supporting this notion. Our studies have demonstrated the similar flow of N6 NA genes of other HxN6 AIVs. Closely related N6 NA genes in clades H and J appeared to circulate among Japan, Mongolia, and Europe and among Mongolia, western Asia, and Europe, respectively (Fig 3e).

Several migratory bird flyways have been considered to contribute to AIV dissemination across Eurasia, and the regions where flyways overlap play important roles as relay points in the spread of viruses [48, 49]. The Central Asia Flyway, the East Africa—Western Asia Flyway, and the East Asia—Australia Flyway overlap in Mongolia and eastern Siberia (Fig 8 purple shade) [47], and the genetic relationship among AIVs isolated along those flyways was evident in clades C, D, E, and F. AIVs that spread from Mongolia to southwestern Siberia, where the Black Sea—Mediterranean Flyway stretches to Europe and West Africa, were also observed in clades C and F. Therefore, AIVs in East Asia could spread to Europe via areas where the Black Sea—Mediterranean Flyway as well as the Central Asia and the East Africa—Western Asia Flyways overlap (Fig 8 green shade), and vice versa. Previous reports highlighted the importance of southern Siberia for the dissemination of AIVs [25, 28, 50, 51]. Lewis et al revealed the dissemination of AIVs from Asian part of southern Siberia to Australia as well as to Europe and Asia by analyzing all segments [50] and the European part of Siberia facing Arctic ocean was estimated to be a relay point of the dissemination of H5N8 HPAIVs from Asia to Europe [25]. On the other hand, few genetic data of AIVs are available from north of southern Siberia, where migratory birds flock for breeding via various flyways, in spite of the higher prevalence of wild birds in breeding areas than in non-breeding areas [48, 52, 53]. Furthermore, the relationship between the migration of birds and the spread of viruses had not been clarified in this area. Dissemination of AIVs between Yakutia and East Asia observed in the present study expand the understanding of the dynamics of AIVs along with previous studies. To further investigate the dynamics of viruses in areas where flyways overlap, it is essential to analyze AIVs as well as movements of birds in northern Siberia.

Overlapping of flyways also plays an important role in the intercontinental movement of AIVs between Eurasia and North America and between Eurasia and Africa. Previous studies revealed the importance of the Alaskan Peninsula, where the East Asia—Australia, the Pacific Americas, and the Mississippi Americas Flyways overlap, and a variety of AIVs were reported (Fig 8 light blue shade) [10, 28, 54]. Along the East Asia—Australia Flyway, some of the Japanese strains were genetically related to AIVs isolated in Alaska, consistent with the above-mentioned report. N6 NA genes from African and European strains comprised clade I and the African strain was isolated from a pelican (Fig 7e). Great white pelicans habitually migrate from Europe to Africa through Israel [55], where the Black Sea—Mediterranean Flyway and the East Africa—Western Asia Flyway overlap. The close relationship between AIVs in Europe and Zambia suggests that European strains are relayed in those regions. Several reports regarding the dissemination of HPAIVs along each flyway have been published [56–59], but there are no such reports regarding overlap of these flyways. The migration flyways described in Fig 8 are only those of shore birds, but there is also a possibility of the direct transmission of AIVs between Europe and Zambia through bird species such as white storks, which breed in Europe and overwinter in southern Africa, including Zambia [60].

There appear to be biases in traits such as host species and HA subtypes of AIVs constituting particular clades. Charadriiformes were clustered together as hosts of clade J (Table 1). Previous BaTS analyses of several HA genes and all internal genes demonstrated that AIVs isolated from gulls cluster together more than those of ducks [61]. The NS gene of H13 and H16 AIVs in clade J was conserved with 98.5% nucleotide identity, and some other internal genes in this clade showed high identity (PB1: 99.2%; NP: 99.0%; MP: 98.5%). The NS gene might be a factor that restricted the Charadriiformes to H13 and H16 AIVs in a particular lineage(s), as a previous report found H13- and H16-specific amino acid signatures mostly in the NS gene, and several were in the NP gene [62]. However, another phylogenetic analysis indicated that the NS gene is not as important as the NP gene for host specificity in H13 AIVs [63]. These results suggest that the gene constellations of H13/H16 AIVs examined in this study could be advantageous in Charadriiformes, resulting in a linkage between certain species and specific genes. It should be noted, however, that biases for targets species, locations, and times in surveillance studies could influence the number of AIVs isolated from specific species, resulting in the possibility to influence the results of BaTs analyses. Additionally, annotation of host species was not well classified in some AIVs used in our analyses; no species were noted in the case of ducks and gulls, and “wild bird” or “wild waterfowl” was used as the host annotation. Detailed identification and registration of host species would help researchers to scrutinize the relationships between viral genes and host specificity.

The map obtained by phylogeographic analysis does not always reflect the actual movements of viruses. Linkages between geographically distant places with blanks for several years, as in clade C (Fig 4c, red type lines), clade E (Fig 4f, red type lines), and clade M (Fig 7e, blue type lines), probably imply that viruses were disseminated via other places where no isolates were reported. For example, in clade M, it is reasonable to consider that dissemination of AIVs between Japan and the north-central United States occurred via the Alaskan Peninsula rather than directly, when we take wild bird migration flyways into account. Another limitation in this study was that only N6 NA gene was phylogeographically analyzed, therefore, actual movement of viruses which involved reassortment events might be overlooked. For further study, phylogeographic analyses based on full genomes of AIVs could improve our understanding of how AIVs are spreading and appropriate setting of the areas for AIV surveillance on the basis of the ecology of wild birds would fill the gap. This in turn would help us to forecast HPAI outbreaks in specified regions.

## Conclusion

Our phylogeographic analysis revealed that intercontinental as well as intracontinental dissemination of AIVs was related to the movement of migratory birds. Overlapping of migration flyways plays an important role in long-distance dissemination of AIVs. Therefore, continuing AIV surveillance in those areas is important for forecasting the spread of these viruses.

## Supporting information

S1 Fig Detail of trees from the entire N6 NA maximum likelihood phylogenetic tree. Identified clades defined on the basis of the rule in this study are green and red, corresponding to the colors in Fig 1. Bootstrap values of 60 or higher are shown.
(TIF)

S2 Fig Detail of trees from the entire N6 NA maximum likelihood phylogenetic tree. Identified clades defined on the basis of the rule in this study are green and red, corresponding to the colors in Fig 1. Bootstrap values of 60 or higher are shown.
(TIF)

S3 Fig Detail of trees from the entire N6 NA maximum likelihood phylogenetic tree. Identified clades defined on the basis of the rule in this study are green and red, corresponding to the colors in Fig 1. Bootstrap values of 60 or higher are shown.
(TIF)

S4 Fig Detail of trees from the entire N6 NA maximum likelihood phylogenetic tree. Identified clades defined on the basis of the rule in this study are green and red, corresponding to the colors in Fig 1. Bootstrap values of 60 or higher are shown.
(TIF)

S5 Fig Detail of trees from the entire N6 NA maximum likelihood phylogenetic tree. Identified clades defined on the basis of the rule in this study are green and red, corresponding to the colors in Fig 1. Bootstrap values of 60 or higher are shown.
(TIF)

S6 Fig Detail of trees from the entire N6 NA maximum likelihood phylogenetic tree. Identified clades defined on the basis of the rule in this study are green and red, corresponding to the colors in Fig 2. Bootstrap values of 60 or higher are shown.
(TIF)

S7 Fig Detail of trees from the entire N6 NA maximum likelihood phylogenetic tree. Identified clades defined on the basis of the rule in this study are green and red, corresponding to the colors in Fig 2. Bootstrap values of 60 or higher are shown.
(TIF)

S1 Table Accession numbers of AIVs isolated in this study. (TIF)

S2 Table Bayes Factors from the place to another in each clade. (XLSX)
