# Supplementary figures and images for: Phylogeographic evidence for the inter- and intracontinental dissemination of avian influenza viruses via migration flyways
Source: PLoS One. 2019 Jun 26;14(6):e0218506. doi: 10.1371/journal.pone.0218506 (PMC6594620; doi:10.1371/journal.pone.0218506)

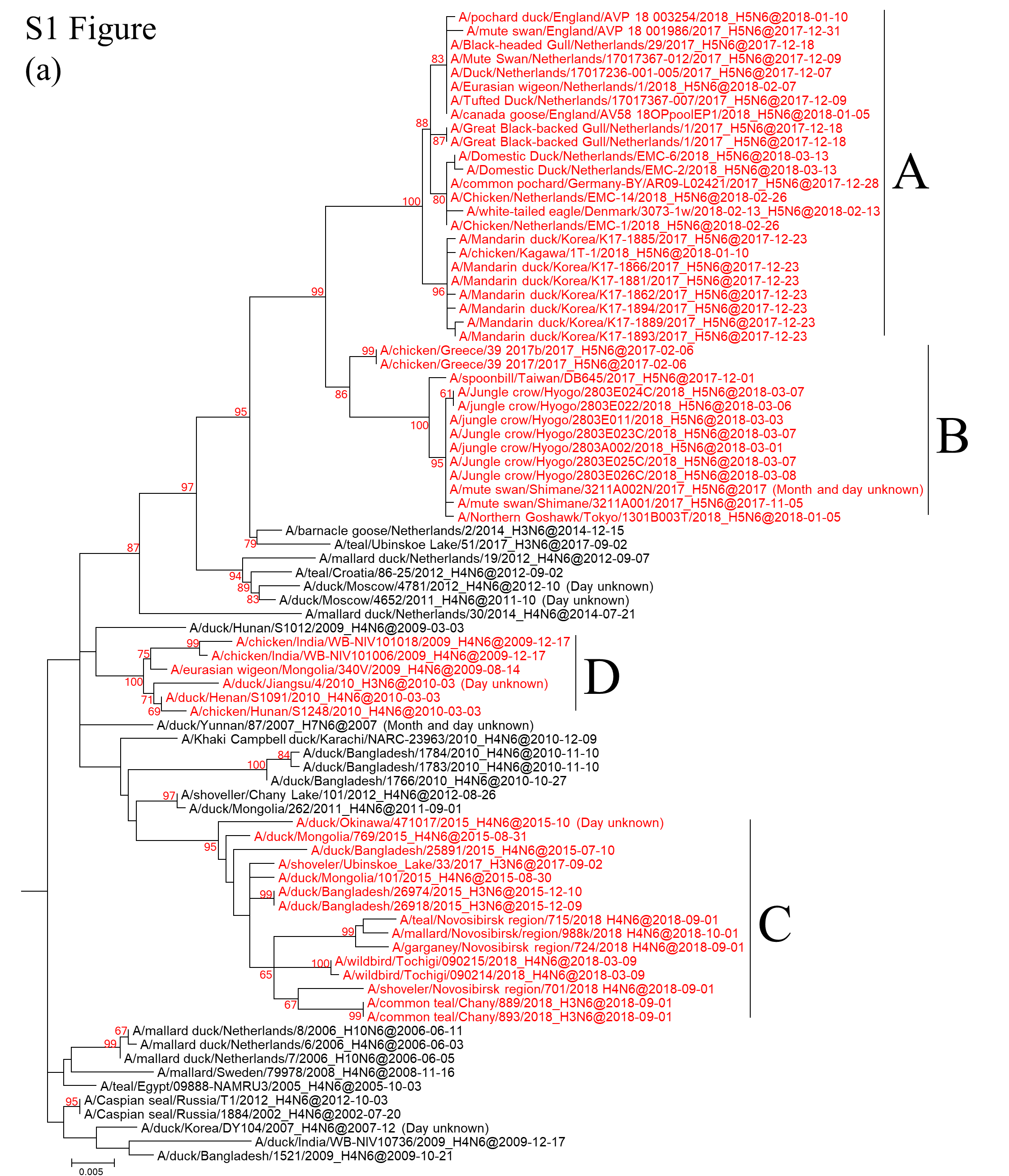

Supplement: S1 Fig — Identified clades defined on the basis of the rule in this study are green and red, corresponding to the colors in Fig 1. Bootstrap values of 60 or higher are shown. (TIF) [file pone.0218506.s001.TIF]

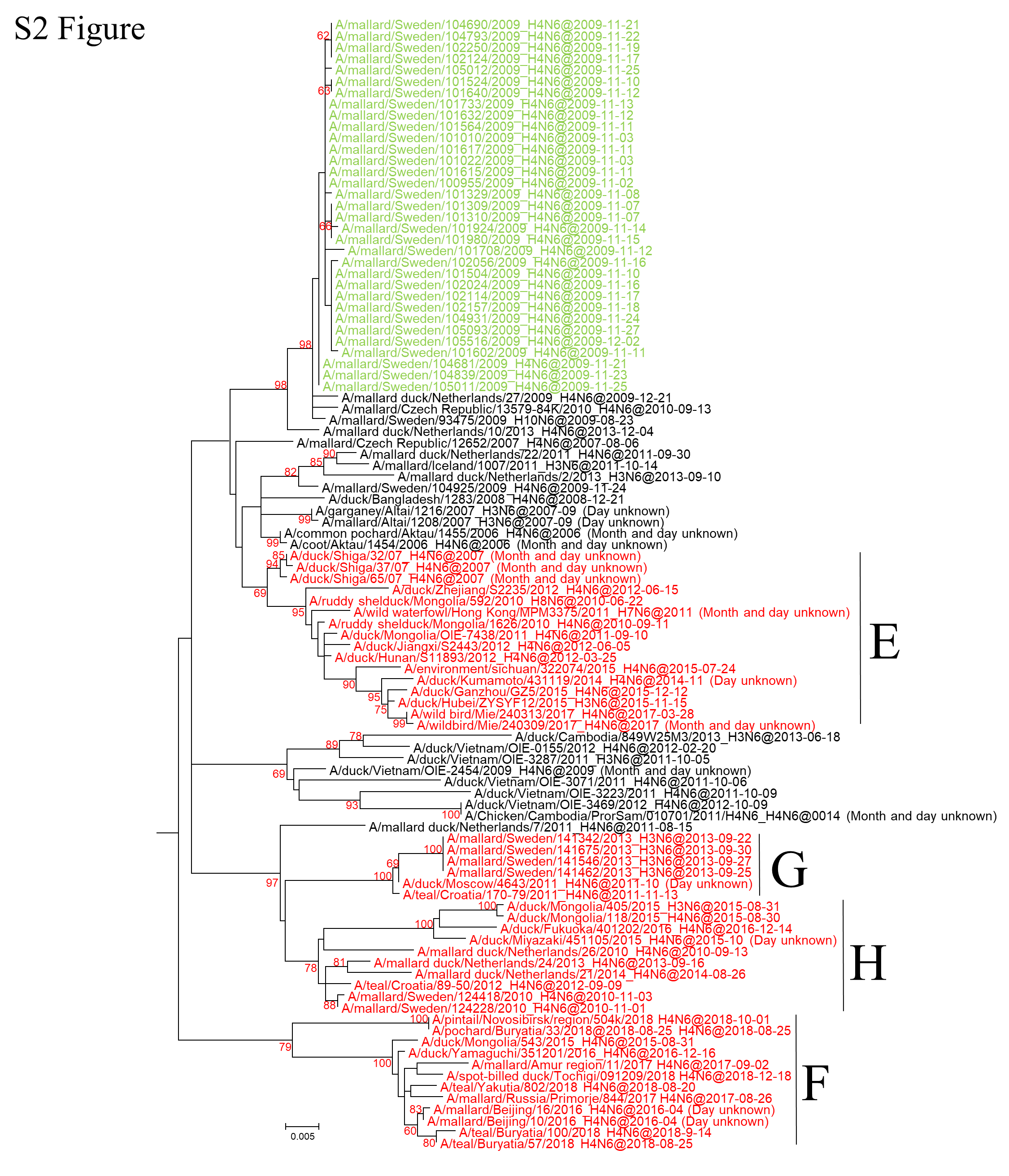

Supplement: S2 Fig — Identified clades defined on the basis of the rule in this study are green and red, corresponding to the colors in Fig 1. Bootstrap values of 60 or higher are shown. (TIF) [file pone.0218506.s002.TIF]

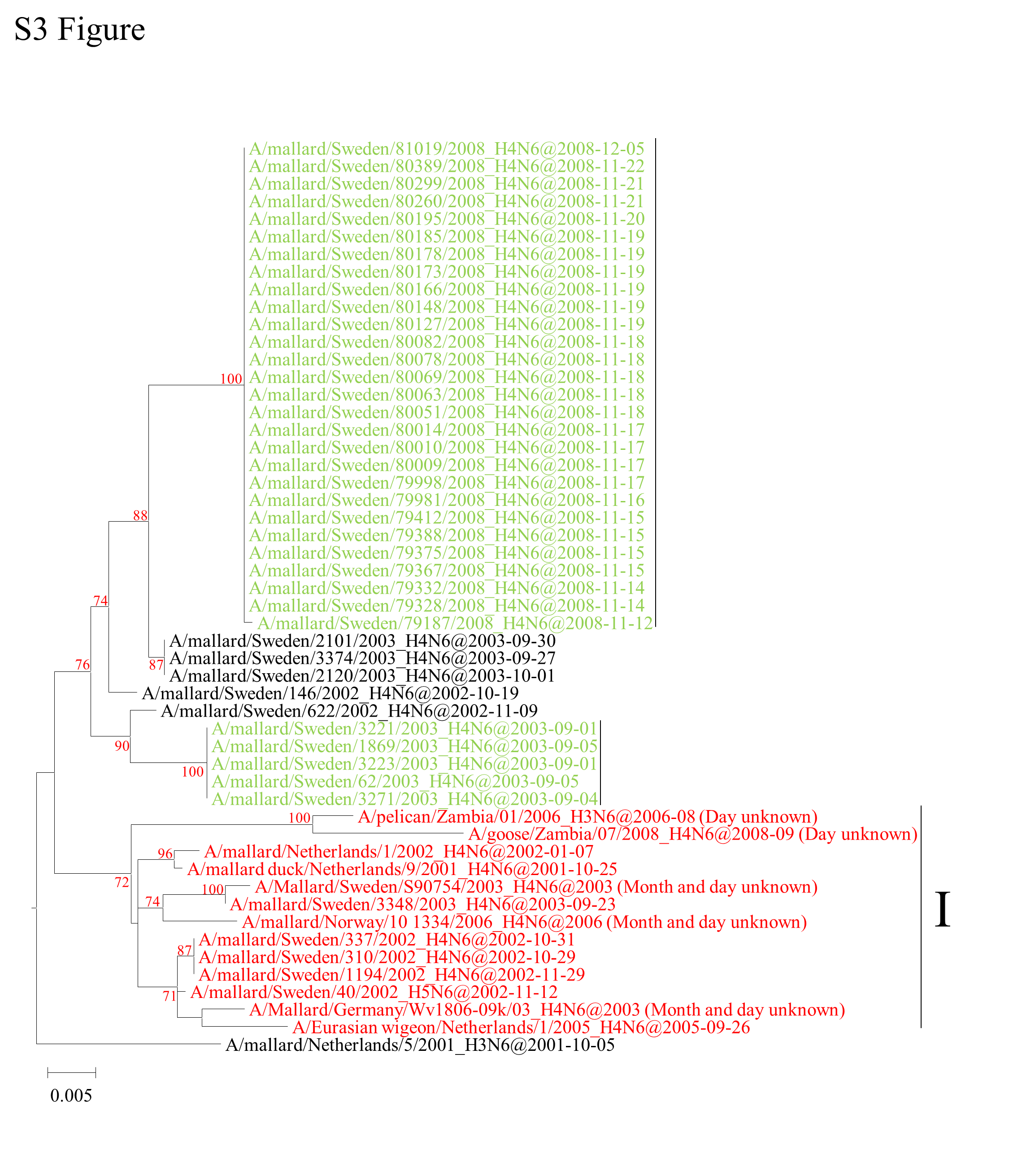

Supplement: S3 Fig — Identified clades defined on the basis of the rule in this study are green and red, corresponding to the colors in Fig 1. Bootstrap values of 60 or higher are shown. (TIF) [file pone.0218506.s003.TIF]

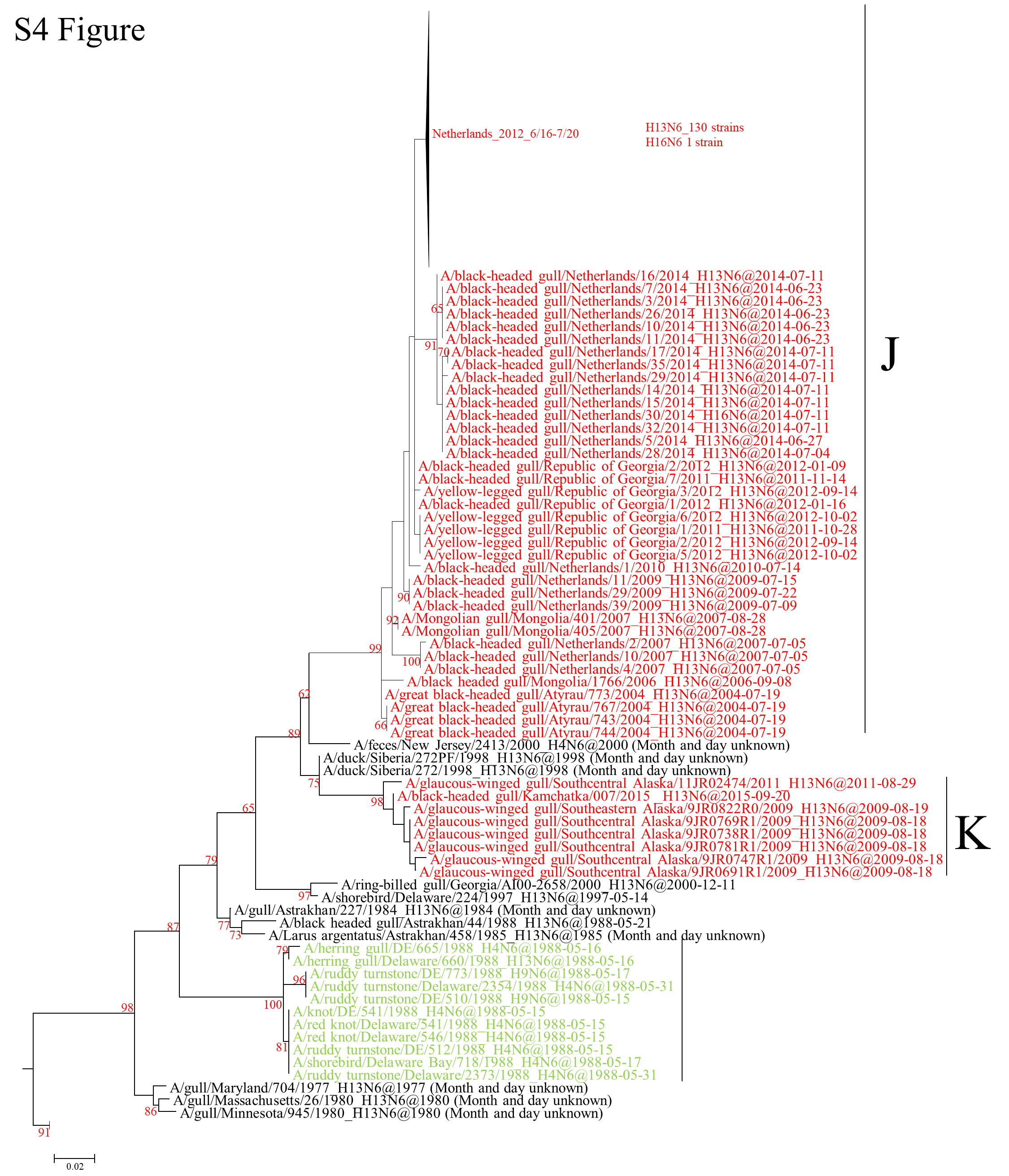

Supplement: S4 Fig — Identified clades defined on the basis of the rule in this study are green and red, corresponding to the colors in Fig 1. Bootstrap values of 60 or higher are shown. (TIF) [file pone.0218506.s004.TIF]

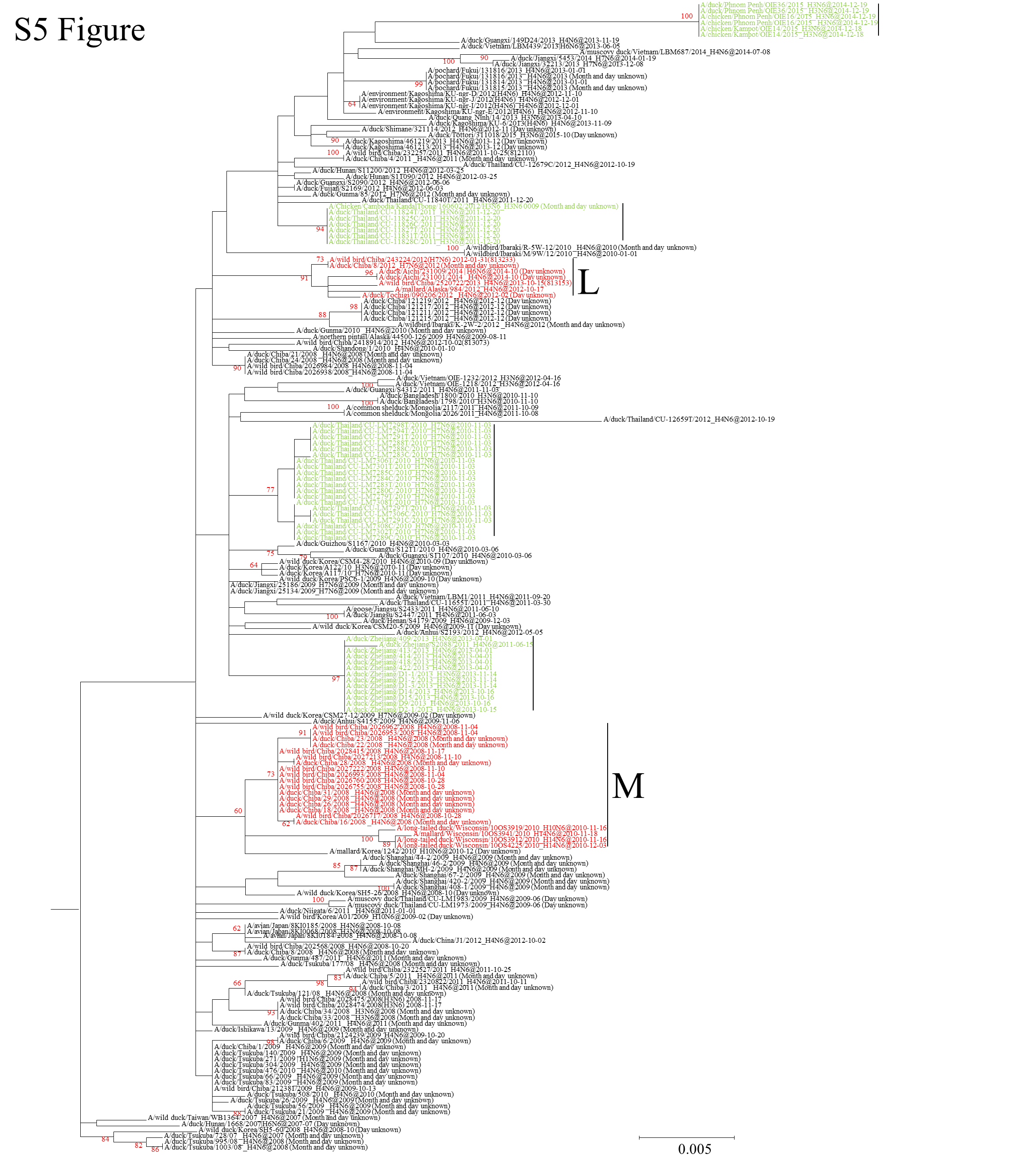

Supplement: S5 Fig — Identified clades defined on the basis of the rule in this study are green and red, corresponding to the colors in Fig 1. Bootstrap values of 60 or higher are shown. (TIF) [file pone.0218506.s005.TIF]

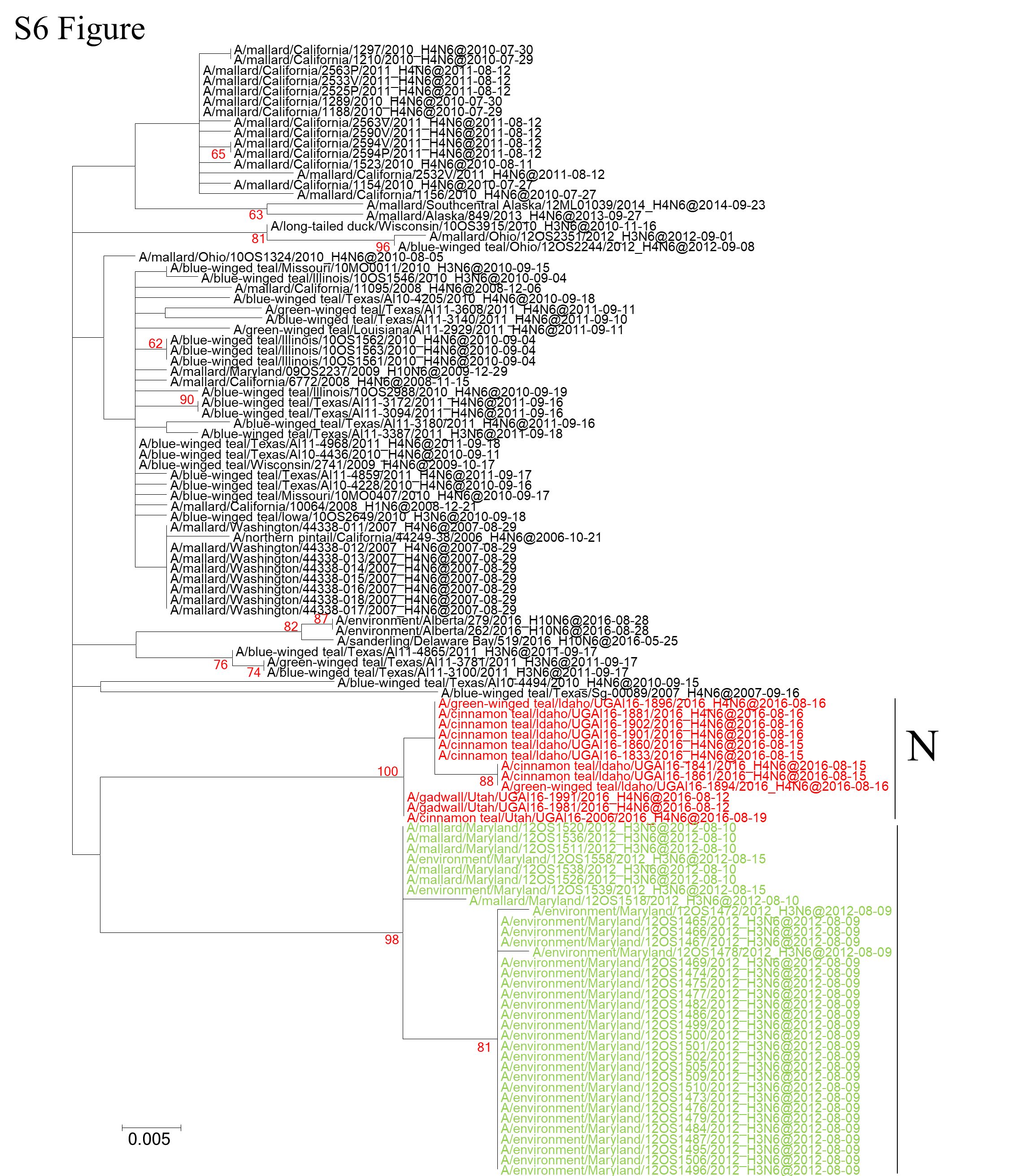

Supplement: S6 Fig — Identified clades defined on the basis of the rule in this study are green and red, corresponding to the colors in Fig 2. Bootstrap values of 60 or higher are shown. (TIF) [file pone.0218506.s006.TIF]

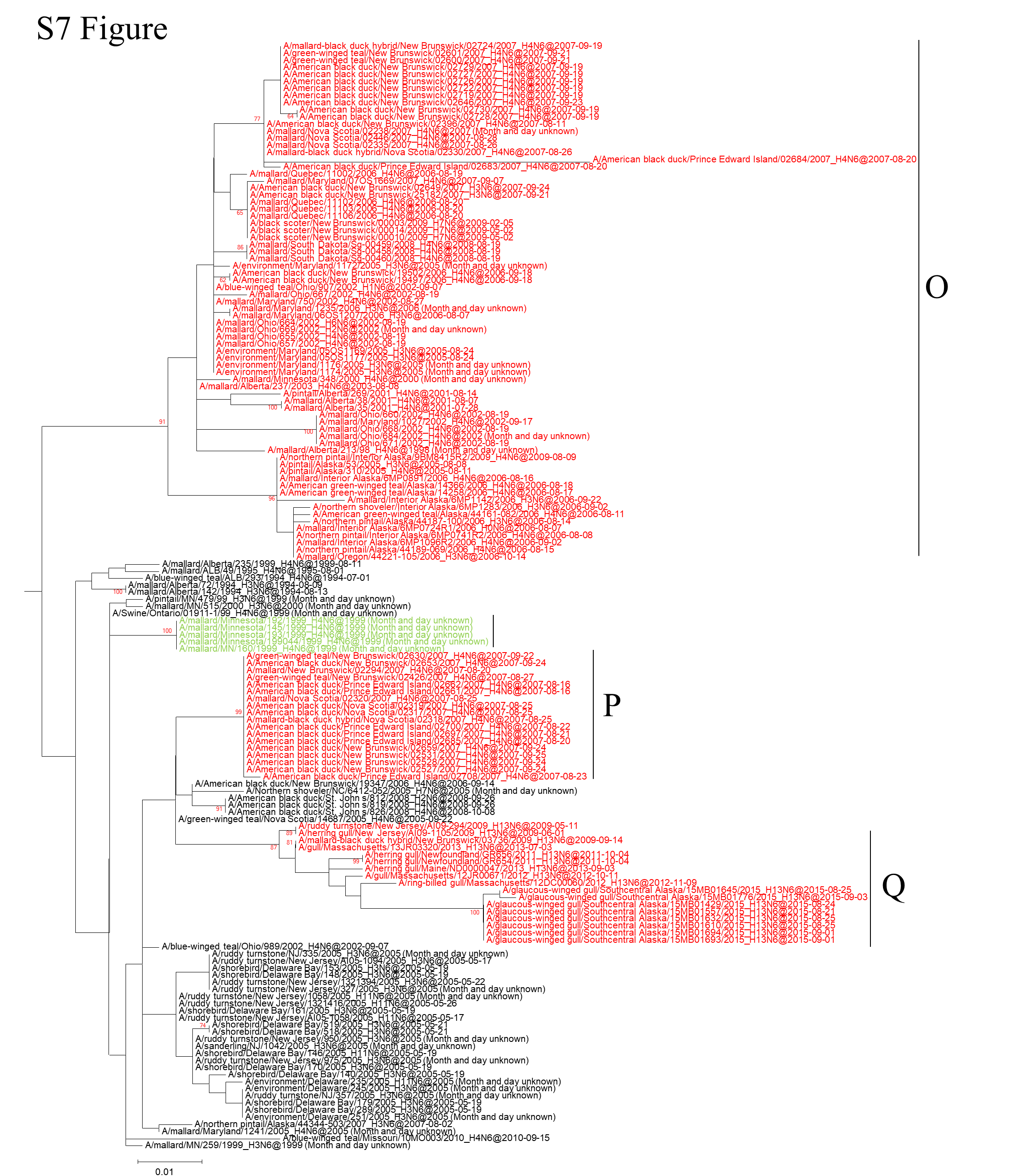

Supplement: S7 Fig — Identified clades defined on the basis of the rule in this study are green and red, corresponding to the colors in Fig 2. Bootstrap values of 60 or higher are shown. (TIF) [file pone.0218506.s007.TIF]

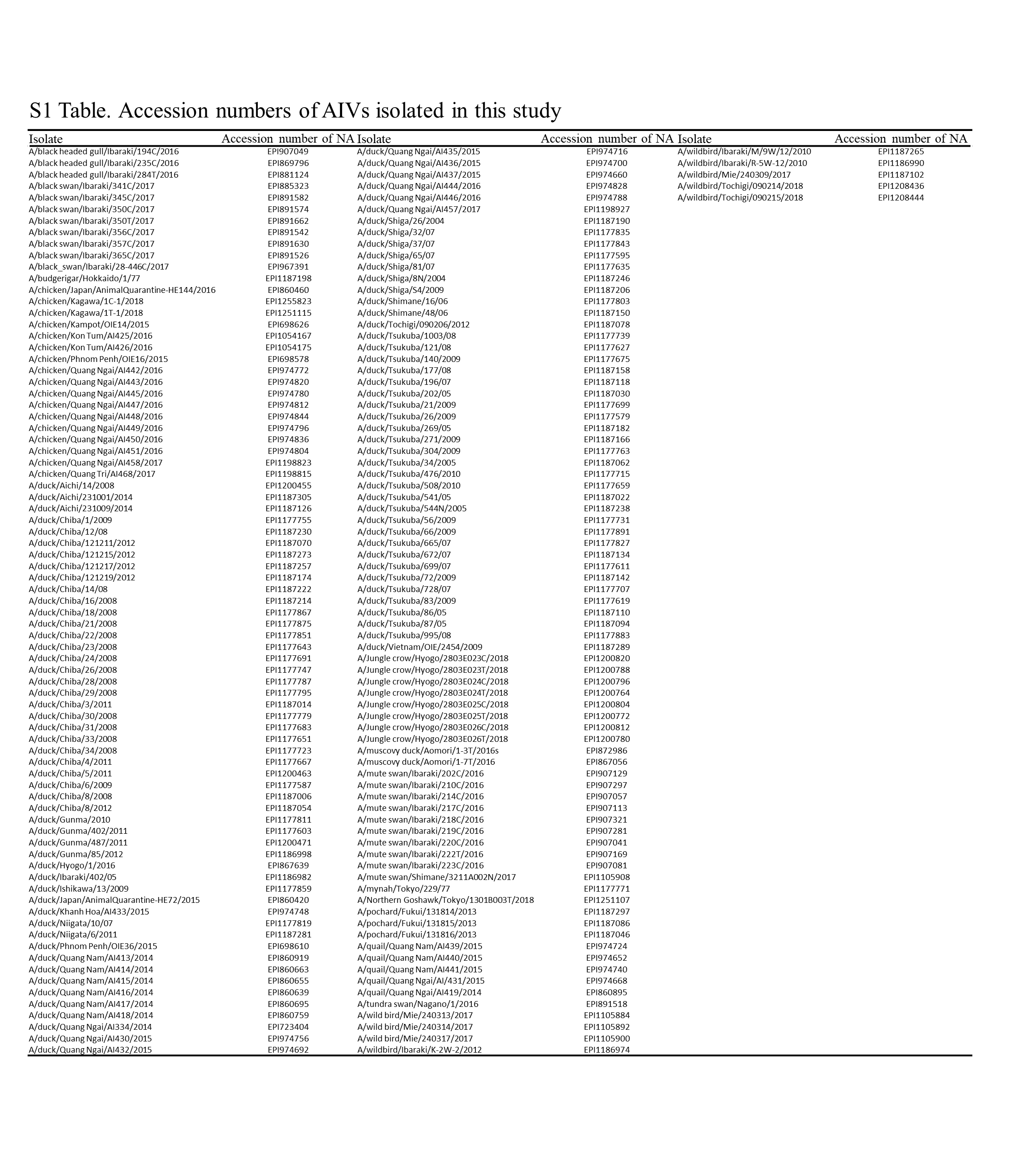

Supplement: S1 Table — (TIF) [file pone.0218506.s008.TIF]
